# Implementation of neoadjuvant immunotherapy in stage III melanoma: a modified Delphi consensus study in a European-accredited cancer center in Ireland

**DOI:** 10.1093/oncolo/oyaf265

**Published:** 2025-08-26

**Authors:** Christopher Cronin, Fiachra Martin, James D Martin-Smith, Nadeem Ajmal, Paul Sullivan, Aileen O’Shea, Muireann Roche, Christian Gulmann, Nazmy Elbeltagi, Barry O’Sullivan, Patrick G Morris, Oscar S Breathnach, Liam M Grogan, Bryan T Hennessy, Adrian Murphy, Megan Greally, Jarushka Naidoo

**Affiliations:** Beaumont Hospital, Dublin, Ireland; Beaumont Hospital, Dublin, Ireland; Beaumont Hospital, Dublin, Ireland; Beaumont Hospital, Dublin, Ireland; Beaumont Hospital, Dublin, Ireland; Beaumont Hospital, Dublin, Ireland; Beaumont Hospital, Dublin, Ireland; Beaumont Hospital, Dublin, Ireland; St Lukes Radiation Oncology Network, Beaumont Hospital, Dublin, Ireland; Beaumont Hospital, Dublin, Ireland; Beaumont Hospital, Dublin, Ireland; Beaumont Hospital, Dublin, Ireland; Beaumont Hospital, Dublin, Ireland; Beaumont Hospital, Dublin, Ireland; Beaumont Hospital, Dublin, Ireland; Beaumont Hospital, Dublin, Ireland; Beaumont Hospital, Dublin, Ireland; RCSI University of Health Sciences, Dublin, Ireland

**Keywords:** neoadjuvant immunotherapy, melanoma, Delphi consensus

## Abstract

**Background:**

The landscape of perioperative immune checkpoint inhibitor (ICI) therapy for stage III melanoma is rapidly evolving. We conducted a modified Delphi consensus process to the define at an institutional level the optimal approach to implementation of a neoadjuvant ICI pathway for melanoma, addressing the themes of patient selection, perioperative therapy, response assessment and operative considerations, and follow-up.

**Methods:**

We developed 28 consensus statements which were circulated to 24 senior members of an institutional melanoma multidisciplinary meeting (MDM) team at the OECI-accredited Beaumont RCSI Cancer Centre, Ireland. Members were invited to anonymously rate statements using a 5-point Likert score. Statements not reaching pre-determined consensus threshold from the initial round of Delphi process would be amended for subsequent rounds.

**Results:**

Two modified Delphi rounds were conducted between May and June 2024, with round 1 results presented locally and at national meeting. Response rates for rounds 1 and 2 were 60% and 46%, respectively. In total, 23 statements of the 28 included (82%) met pre-determined criteria for consensus. Areas where lack of consensus was identified included the use of ICIs to down-stage unresectable disease, response-adapted approaches to adjuvant therapy and the optimal extent of nodal resection.

**Conclusions and Revelance:**

Our process identified important knowledge gaps regarding the multidisciplinary care of stage III melanoma. The statements generated will be used to develop a local pathway for the implementation of neoadjuvant immunotherapy in melanoma, with plans to further expand the Delphi process to other Irish institutions incorporating up to date published data to refine recommendations.

Implications for Practice Our modified Delphi process and multidisciplinary appraisal have allowed for the identification of key knowledge gaps in the rapidly evolving field of neoadjuvant and perioperative therapy in resectable stage III melanoma. Through the contextualization of available data and presentation of important open questions in this dynamic space, our consensus statements and literature review may help to inform the development of multidisciplinary care pathways for the management of resectable node-positive melanoma.

## Introduction

Approximately 330 000 new cases of cutaneous melanoma are diagnosed globally each year, representing 1.7% of all invasive malignancies and accounting for 0.6% of cancer-associated deaths.^1^ In Ireland, more than 1100 new cases are diagnosed annually, with more than 150 associated deaths.^2^ For resectable cutaneous melanoma, melanoma-specific survival rates are closely linked with disease stage. In patients with resected stage III disease, recurrence rates of up to 70% have been historically observed, mostly occurring within the first 2 years of resection.^3^ Immune checkpoint inhibitors (ICIs) targeting PD-1 and CTLA-4, as well as targeted therapies such as BRAF/MEK inhibitors, have transformed the landscape of advanced or unresectable melanoma wherein 5-year survival rates of 30%-50% have been achieved in phase III clinical trials.^4–6^ More recently, the LAG-3-targeting ICI relatlimab has also demonstrated clinical benefit when combined with PD-1 blockade in patients with advanced melanoma, based on a phase III trial.^7^ Multiple phase III randomized controlled trials (RCTs) of ICIs in the adjuvant setting have demonstrated significant improvements in recurrence-free survival (RFS) and distant metastasis-free survival.^8^^,^^9^ Based on these data, adjuvant ICI monotherapy has emerged as a standard of care for patients with resected stage III disease including stage IIIA disease with >1 mm involvement in a sentinel node, and is broadly supported by clinical practice guidelines.^10–13^

Despite the benefit afforded by adjuvant ICI therapy in resected stage III melanoma, approximately 30% of patients develop disease recurrence within 2 years of completing adjuvant therapy.^8^^,^^9^ ICIs in the neoadjuvant setting offer the potential for enhanced antitumor immunity which may improve long-term survival for resectable disease. Adopting a neoadjuvant approach also affords the potential to stratify patients based on pathological response.^14^ Multiple phase II trials have assessed the safety, and efficacy of neoadjuvant ICIs in resectable stage III melanoma.^15^ In the OpACIN-neo trial, patients with resectable, clinically detectable stage III melanoma were randomly assigned to receive neoadjuvant ipilimumab plus nivolumab following 3 different dosing schedules with the aim of identifying the optimal neoadjuvant combination. A pathologic complete response (pCR) was observed in 57% of patients who received 2 cycles of ipilimumab (1 mg/kg) and nivolumab (3 mg/kg) given on a 3-weekly schedule. The estimated 3-year RFS for all included patients was 82%.^16^ The phase II PRADO study aimed to explore a response-adapted approach to perioperative therapy. Patients received 2 cycles of nivolumab with ipilimumab, followed by resection of an index lymph node (ILN). Patients who experienced a major pathological response (mPR) did not undergo total lymph node dissection (TLND) or receive further systemic therapy, patients with partial response underwent TLND but no further systemic therapy, and patients with pathologic non-response (pNR) underwent TLND and received adjuvant therapy. The 2-year RFS rate for patients who achieved mPR with neoadjuvant therapy was 93%.

The largest phase II study (SWOG S1801) randomized patients with clinically detectable, resectable stage IIIB-IVC melanoma, to receive three doses of neoadjuvant pembrolizumab followed by surgery and fifteen doses of adjuvant pembrolizumab to complete 1 year of pembrolizumab in total, versus upfront surgical resection followed by up to 1 year of adjuvant pembrolizumab.^17^ In this study, the landmark 2-year event-free survival (EFS) analysis demonstrated significantly higher 2-year EFS rates in the neoadjuvant-adjuvant group compared to the adjuvant-only group (72% vs 49%). Neoadjuvant therapy was associated with pathological complete response in 21% of patients. The more recently reported phase III NADINA trial randomized patients to upfront (TLND) followed by adjuvant nivolumab versus combined ipilimumab with nivolumab administered 3-weekly for a total of 2 cycles prior to TLND. Adjuvant therapy in the neoadjuvant arm was selected based on pathologic response with patients experiencing pCR or mPR proceeding to surveillance only without further systemic therapy. Patients who did not experience a pCR or mPR following neoadjuvant therapy, received an additional 11 doses of 4-weekly nivolumab schedule to complete one year in total. Adjuvant BRAF-targeted therapy for the same duration was permitted for patients with melanoma harboring a BRAF V600 mutation. The study demonstrated significant improvements in 12-month EFS in the neoadjuvant arm (83.7% vs. 57.2%).^18^

As the evidence base supporting the use of neoadjuvant therapy in clinical stage III melanoma evolves, recommendations from international guidelines vary with respect to the optimal perioperative systemic approach for this patient population.^10–13^^,^^19^ A challenge for multidisciplinary teams involved in the care of patients with melanoma lies in modifying local practices to align with the rapidly evolving evidence-base, while maintaining standards and consistency in care pathways and service provision. Within this context, we undertook a modified Delphi consensus project to define at an institutional level:

1. A series of consensus statements to inform the implementation of a neoadjuvant immunotherapy pathway for locally advanced melanoma.
2. A summary of the available and emerging evidence supporting neoadjuvant ICIs in resectable melanoma.
3. The practical application of a neoadjuvant ICI pathway in an Irish healthcare setting.
4. A consensus and evidence-based new standard neoadjuvant pathway for the care of patients with locally advanced melanoma, through multidisciplinary collaboration.

## Methods

A literature review was conducted including a comprehensive search of PubMed, and Medline for published literature relevant to neoadjuvant immunotherapy in melanoma. English language articles published between January 2004 and April 2024 were included. Bibliographies and relevant conference proceedings were also reviewed. Following literature review, a draft set of consensus statements following the PICO format (population, intervention, comparison, and outcome) was generated with input and modifications from senior members of an institutional melanoma multidisciplinary meeting (MDM) team at the OECI-accredited Beaumont RCSI Cancer Centre, Dublin, Ireland, including medical oncology, radiation oncology, plastic surgery, dermatology, histopathology, and radiology.^20^ In total, 28 statements were composed exploring the following 4 themes:

1. Staging and patient selection
2. Perioperative therapy
3. Response assessment and operative considerations
4. Surveillance and follow-up

Once the statements were developed, an online modified Delphi process was conducted during which the statements were circulated to relevant specialists in the MDM listed above. MDM members were invited to anonymously rate statements using a 5-point Likert score (1: strongly agree, 2: agree, 3: neither agree nor disagree, 4: disagree, and 5: strongly disagree). Respondents were also invited to make specific comments on statements in a free-text box. Consensus was defined as 75% or more of responses falling within the “strongly agree” or “agree” Likert score range. Statements not reaching consensus threshold from the initial round of Delphi process would be iteratively updated for subsequent rounds and discussed at an in-person meeting to describe opinions and outcomes.

## Results

### Conduct of modified Delphi process

In total, two modified Delphi rounds were conducted. The first round was held in April 2024 following which results were presented to the institutional melanoma MDM and a national melanoma MDM. Feedback from these meetings was used to modify the consensus statements, and a second Delphi round was conducted in June 2024. Only statements that did not meet consensus during the first round were included for the second round. For each Delphi round, 24 senior members of our institutional melanoma MDM were invited. All invited participants worked as fully accredited consultants (attending equivalent) in their respective fields, and were regularly involved in the diagnosis and management of patients with melanoma as part of the institutes MDM. Of the 24 invited participants, 7 (29%) were medical oncologists, a further 7 (29%) were plastic/reconstructive surgeons, 5 (21%) were dermatologists, 3 (13%) were pathologists, and 1 representative was included from the specialities of radiation oncology and radiology, (4%, respectively). Fourteen responses were received for the first Delphi round (60% response rate), and 11 completed responses for the second round (46% response rate). Consensus was achieved for 22 of 28 statements (79%) circulated as part the first Delphi round. Of the 6 statements in which consensus was not achieved, consensus was achieved for 1 of these statements in the subsequent Delphi round. In total, 23 statements of the 28 included (82%) met pre-determined criteria for consensus. Consensus statements are outlined in Tables 1-4. Table 5 describes the percentage of responses to each group of statements which met the criteria for consensus across the two Delphi rounds and the changes in level of agreement achievement between rounds. Selected results are presented within each of the four themes, alongside relevant findings from the literature review.

**Table 1.** Statements regarding staging and patient selection.

Statement	Consensus achieved
**Statement 1: For patient with histologically confirmed stage III melanoma being considered for curative surgical resection, baseline staging investigations should include contrast-enhanced CT based on anatomical location of disease, whole body FDGPET/CT, and contrast-enhanced MRI brain.**	Yes
**Statement 2: Patients with resectable melanoma potentially suitable for perioperative immunotherapy should be reviewed at a dedicated multidisciplinary meeting with representation from relevant specialties once staging investigations have been performed.**	Yes
**Statement 3: Suitability for definitive surgical resection should be established prior to the commencement of neoadjuvant systemic therapy based on a dedicated multidisciplinary meeting discussion.**	Yes
**Statement 4: Suitability for resection should account for patient factors such as performance status and co-morbidities, as well as disease factors including anatomical location of disease and feasibility of achieving an R0 resection based on pre-operative imaging and clinical assessment.**	Yes
**Statement 5: Neoadjuvant systemic therapy should be considered for patients with resectable stage IIIB-IIID disease with one or more clinically detectable nodal metastasis.**	Yes
**Statement 6: For patients with locally advanced or metastatic melanoma deemed to be unresectable based on initial staging investigations and clinical assessment, routine use of neoadjuvant systemic therapy to facilitate definitive surgical resection is not recommended.**	No
**Statement 7: Upfront systemic therapy should be considered for patients with limited in-transit metastases, satellite metastases, or oligometastatic disease. Definitive resection of residual disease following delivery of systemic therapy may be considered provided surgical resection without unacceptable morbidity is feasible.**	Yes

**Table 2.** Statements regarding perioperative therapy.

Statement	Consensus achieved
**Statement 8: For patients with clinically detectable stage III melanoma for whom neoadjuvant systemic therapy is being considered, the preferred agent is pembrolizumab given on a 3-weekly schedule for 3 cycles pre-operatively, followed by a further 15 cycles in the adjuvant setting to complete 1 year total of systemic therapy.**	Yes
**Statement 9: For patients with clinically detectable stage III melanoma harboring a BRAF V600 mutation, neoadjuvant systemic therapy with BRAF-targeted therapy for a duration of 8-12 weeks may be considered as an alternative to immunotherapy.**	Yes
**Statement 10: Patients should be informed that IrAEs from neoadjuvant immunotherapy may be severe and could impact on the timing of conduct of surgical resection.**	Yes
**Statement 11: For patient who received 3 cycles of pembrolizumab in the neoadjuvant setting, a further 15 cycles of pembrolizumab should be administered following surgical resection, regardless of pathological response to neoadjuvant therapy.**	Yes
**Statement 12: Patients selected for adjuvant systemic therapy after neoadjuvant therapy should commence treatment within 12 weeks of surgical resection.**	Yes
**Statement 13: Omission of adjuvant therapy may be considered if severe irAEs complicate neoadjuvant immunotherapy or significant surgical complications arise following definitive resection.**	Yes
**Statement 14: A modified approach involving de-escalation or omission of adjuvant systemic therapy if a pathological complete response or major pathological response is achieved following neoadjuvant therapy may be considered on a case-by-case basis.**	No
**Statement 15: Adjuvant radiotherapy is not recommended following definitive surgical resection, particularly when perioperative systemic therapy is delivered. It may be considered for patients at high risk of local recurrence after neoadjuvant systemic therapy (eg, lymph node diameter >3 cm, multiple metastatic lymph nodes, or the presence of extracapsular nodal extension) who are not suitable for adjuvant systemic therapy.**	Yes

Abbreviation: IrAEs, immune-related adverse events.

**Table 3.** Statements regarding response assessment and operative considerations.

Statement	Consensus achieved
**Statement 16: Radiologic response to neoadjuvant systemic therapy should be assessed with contrast-enhanced CT based on the site of disease 2-4 weeks post completion of neoadjuvant therapy.**	Yes
**Statement 17: Assessment of radiologic response to systemic therapy may require additional cross-sectional imaging including ultrasound and MRI based on anatomical location of disease to guide operative management.**	Yes
**Statement 18: Surgical resection should take place within 4-6 weeks of completion of neoadjuvant systemic therapy.**	Yes
**Statement 19: Total lymph node dissection should remain the standard surgical approach for clinically detectable stage III melanoma regardless of radiological or pathological response to neoadjuvant therapy.**	No
**Statement 20: Consideration may be given to more limited, selective dissection of involved nodal disease following neoadjuvant systemic therapy based on anatomical location and potential surgical morbidity on a case-by-case basis.**	Yes
**Statement 21: A modified surgical approach following neoadjuvant systemic therapy should only be considered following informed discussion with the patient regarding potential risks and limitations pre-operatively.**	Yes
**Statement 22: Pathological assessment of resection specimens including assessment of pathological response to neoadjuvant therapy should be carried out in line with the International Neoadjuvant Melanoma Consortium consensus recommendations. Evidence of therapy-specific effects should be described and quantified, including the relative percentage of tumor bed occupied by viable tumor, tumoral necrosis and/or melanosis, inflammatory infiltrate, and fibrosis.**	Yes
**Statement 23: Pathological response to neoadjuvant therapy should be defined as follows: pCR: no viable tumor is evident in the treated tumor bed; mPR: <10% viable tumor remains in treated tumor bed; partial pathological response (pPR): 10%-50% viable tumor is present in the treated tumor bed.**	Yes

Abbreviations: mPR, major pathological response; pCR, pathological complete response; pPR, partial pathological response.

**Table 4.** Statements regarding surveillance and follow-up.

Statement	Consensus achieved
**Statement 24: Patient follow-up following definitive surgical resection of stage III/IV disease should include clinical assessments (skin examination and assessment of lymph node basins) every 4-6 months for the first 3 years after surgical resection, then every 6 months for years 4 and 5.**	Yes
**Statement 25: For patients with resected stage IIIA-C melanoma, radiologic imaging including whole body PET/CT and MRI brain should be performed every 6 months for the first 3 years after resection, then every 12 months for years 4 and 5.**	No
**Statement 26: For patients with resected stage IIID-IV melanoma, radiologic imaging including whole body PET/CT and MRI brain should be performed every 3 months for the first 3 years after resection, then every 6 months for years 4 and 5.**	No
**Statement 27: Where PET/CT is not available for the surveillance of resected stage III-IV melanoma, contrast-enhanced CT TAP may be used as an alternative. For patients with melanoma involving the head and neck for whom whole body PET/CT is not performed, cross-sectional imaging should include contrast-enhanced CT neck and TAP.**	Yes
**Statement 28: For patient with stage III melanoma with a positive sentinel lymph node that have not had a complete lymph node dissection and are not undergoing PET/CT or contrast-enhanced CT surveillance imaging, ultrasound of the relevant nodal basin should be considered every 4 months for years 1-3, then every 6 months for years 4 and 5.**	Yes

**Table 5.** Consensus achieved for included statements.

	Level of consensus round 1 (%, *n* = 14)	Level of consensus round 2 (%, *n* = 11)
**Staging and patient selection**		
** Statement 1**	100	
** Statement 2**	100	
** Statement 3**	100	
** Statement 4**	100	
** Statement 5**	86	
** Statement 6**	57	73
** Statement 7**	79	
**Perioperative therapy**		
** Statement 8**	71	82
** Statement 9**	79	
** Statement 10**	93	
** Statement 11**	79	
** Statement 12**	93	
** Statement 13**	93	
** Statement 14**	57	63
** Statement 15**	79	
**Response assessment and operative considerations**		
** Statement 16**	93	
** Statement 17**	86	
** Statement 18**	100	
** Statement 19**	71	73
** Statement 20**	86	
** Statement 21**	86	
** Statement 22**	93	
** Statement 23**	86	
**Surveillance and follow-up**		
** Statement 24**	86	
** Statement 25**	64	55
** Statement 26**	64	55
** Statement 27**	79	
** Statement 28**	100	

### Staging and patient selection

#### Defining resectability in a neoadjuvant pathway

High levels of consensus were achieved for statements 2-4 which recommend dedicated MDM review with the goal of establishing resectability prior to entering a neoadjuvant pathway. Surgical resectability should be established upfront, accounting for patient factors such as medical co-morbidities and operative risk, as well as disease factors including anatomical location of disease and feasibility of achieving an R0 resection.

A standardized definition of resectability in stage III melanoma has not been clearly delineated and may vary based on institutional practice and other factors. In the SWOG S1801 study, resectability was not defined beyond staging eligibility criteria and local MDM determination of resectability.^17^ The phase III NADINA trial included patients with a limited number of in-transit metastases (up to 3 resectable lesions), but did not further define resectability beyond staging eligibility criteria.^18^ In other phase II studies of neoadjuvant ICI therapy in resectable stage III/IV melanoma, resectability was determined by local MDM conference consensus.^16^^,^^21^^,^^22^ The International Neoadjuvant Melanoma Consortium (INMC) recommends that determining resectability should account for anatomical ability to achieve an R0 resection, perioperative risk, surgical morbidity, and likelihood of early recurrence, categorizing cases as resectable, technically unresectable, and unresectable due to likely futility or unacceptable morbidity.^23^

#### Patient selection for a neoadjuvant approach

Statement 5 proposes that neoadjuvant immunotherapy be considered for patients with IIIB-IIID melanoma with one or more clinically detectable nodal metastases, based on consensus reached in 86% of respondents. The definition of “clinically detectable” stage IIIB-IIID melanoma varied slightly between the SWOG S1801 and NADINA trials. In SWOG S1801, clinically detectable was defined as a disease that is apparent on physical examination or radiographic imaging (radiologically measurable lymph nodes of ≥1.5 cm in short axis, and/or other disease site if ≥1.0 cm on CT/MRI).^17^ In the NADINA trial, clinically detectable lymph nodes were defined as palpable on clinical examination, ≥1.5 cm on cross-sectional imaging, or a PET/CT positive and biopsy-proven lymph node of any size.^18^

Statement 7 proposes upfront systemic therapy for patients with limited in-transit and satellite metastases, and oligometastatic disease, followed by consideration of surgical resection of measurable disease. While consensus was achieved for this statement with 79% of respondents agreeing with its content, lower levels of consensus were achieved compared with other statements in this section. The optimal management of patients with resectable oligometastatic and in-transit metastases in the era of neoadjuvant therapy remains unclear, given the paucity of high-level evidence for this patient population. Phase II studies in this space variably permitted the inclusion of patients with limited oligometastatic and in-transit disease.^16^^,^^21^^,^^22^^,^^24^ The SWOG S1801 study permitted the inclusion of any in-transit and oligometastatic disease deemed resectable at local MDT conference, except for brain metastases, representing 14% of patients in the neoadjuvant arm.^17^ The NADINA trial excluded patients with stage IV disease but did allow the inclusion of patients with up to 3 resectable in-transit metastases—representing approximately 10% of patients in this study.^18^

#### Systemic therapy and cytoreduction of unresectable disease

Statement 6 addresses the question of cytoreduction to facilitate definitive resection of disease deemed unresectable on initial presentation. This statement advises against routine use of ­neoadjuvant therapy to downstage disease deemed initially unresectable. Iterative changes to the wording of this statement between Delphi rounds did increase the proportion of respondents in agreement from 57% to 73%, but the pre-defined criteria for consensus were ultimately not met.

SWOG S18101 and NADINA limited inclusion to patients deemed resectable on initial presentation. Similarly, phase II studies assessing neoadjuvant BRAF-targeted therapy limited inclusion to resectable disease.^25^^,^^26^ Two small, early-phase studies have explored the use of neoadjuvant targeted therapy in unresectable or borderline-resectable. The phase II single-arm REDUCTOR trial assessed the feasibility of cytoreduction with neoadjuvant BRAF/MEK-inhibitor to facilitate surgical resection of locally advanced, unresectable BRAF-mutant melanoma. Radiological response was observed in 17/21 patients (81%) following 8 weeks of Dabrafenib and Trametinib therapy, and R0 resection was achieved in 17/21 patients (81%).^27^ A prospective, single-center non-randomized study explored the efficacy of neoadjuvant targeted therapy for patients with borderline resectable BRAF-mutant IIIB-IV melanoma. All 47 patients included in the study proceeded to surgical resection, with an R0 resection rate of 87%. While these data offer some support to this strategy, the lack of randomized data led to the development of our consensus statement in its current form.

### Perioperative therapy

#### Selection of regimen for neoadjuvant systemic therapy

Consensus was achieved for statement 8 which recommends neoadjuvant and adjuvant pembrolizumab, informed by the randomized phase II SWOG S1801 study as the preferred perioperative systemic therapy regimen.^17^ While predetermined levels of consensus were not met with round 1 of our Delphi process for this statement (71%), MDM group discussion of reported data and approved perioperative systemic treatment options led to 82% consensus in round 2 without changes to the wording of the statement. Consensus was also achieved for statement 9, which proposes BRAF-targeted therapy as an alternative neoadjuvant treatment for resectable stage IIIA-IIID melanoma harboring a BRAF V600 mutation for whom ICI therapy is contra-indicated.

At the time of conducting the Delphi process, SWOG S1801 was the only reported prospective, randomized study assessing neoadjuvant immunotherapy in this setting, with the phase III NADINA trial published subsequently. This approach has not been granted regulatory approval by the European Medicines Agency (EMA) but is approved for reimbursement by the National Cancer Control Programme in Ireland.^28^

Statement 9 allows for the use of BRAF-inhibitor therapy in the perioperative setting in cases where ICI therapy may be contraindicated, an approach supported by the phase II trial data. In the phase II, single-arm NeoCombi study, patients with resectable BRAF-mutated stage IIIB-C melanoma received 12 weeks of neoadjuvant targeted therapy with Dabrafenib plus Trametinib followed by surgical resection and a further 40 weeks of adjuvant therapy to complete 1 year in total. Of the 35 enrolled patients, pCR was observed in 49%.^25^ A single-center phase II study published by Amaria et al. randomized patients with resectable stage IIIB-IV BRAF-mutated melanoma to undergo upfront surgery and adjuvant BRAF-targeted therapy versus 8 weeks of neoadjuvant BRAF/MEK-targeted therapy followed by surgical resection and a further 44 weeks of adjuvant therapy to complete 1 year. Pathological complete response was observed in 7 of the 12 patients (58%) randomized to neoadjuvant targeted therapy, and significant improvements in EFS were observed in the neoadjuvant group.^26^ In neoadjuvant trials of targeted therapy in high-risk resectable melanoma, higher pCR and mPR rates have been observed when compared with neoadjuvant ICI trials in a similar patient population.^14^ However, pooled analysis of neoadjuvant trials in melanoma report that 3-year RFS rates observed with targeted therapy (even when pCR or mPR are achieved) are inferior to neoadjuvant ICI therapy when a similar response is observed, raising the question of durability of remission in high-risk resected melanoma with targeted therapy versus ICI therapy.^29^ Regardless, neoadjuvant BRAF-targeted therapy remains a valid, guideline endorsed treatment option for patients with clinical stage III, BRAF-mutated melanoma and may be offered as an alternative to ICI therapy.

#### Addressing safety concerns and risk of disease progression with a neoadjuvant approach

A further consideration in the implementation of a neoadjuvant pathway is the potential risk of severe irAEs or progression events that may impact the ability to proceed to curative surgical resection. High levels of consensus were achieved for statement 10 which discusses careful counselling of patients regarding these potential risks prior to undertaking neoadjuvant therapy. Similarly, statement 13 which allows for the omission of adjuvant ICI therapy in the event of significant immune-related adverse events during neoadjuvant therapy achieved high levels of consensus with the MDM group.

In SWOG S1801 and NADINA, grade 3-4 irAEs from neoadjuvant ICI were observed in 7%^17^ and 29.7% of patients, respectively.^17^^,^^18^ Despite this, the proportion of patients not proceeding to surgical resection was relatively low. In the SWOG S1801 study, 17/144 (12%) of patients did not proceed to surgical resection, due to disease progression in 12 cases (8%) and immune-related toxicity in 1 patient (1/144; <1%). In the NADINA trial, surgery was not performed due to disease progression in 5 patients (3%), and treatment-associated toxicity in 3 patients (1%).^18^

#### Response-adapted approaches to adjuvant systemic therapy

Consensus was achieved for statement 11, with 79% of respondents agreeing adjuvant therapy should not be modified or de-escalated based on pathological response from neoadjuvant therapy. Statement 14, which allows for de-escalation or omission of adjuvant therapy based on pathologic response on a case-by-case basis, did not meet criteria for consensus, with only 63% of respondents in agreement following the second Delphi round (see Table 5).

Response-adopted omission of adjuvant therapy based on response to neoadjuvant therapy was first explored prospectively in the phase II PRADO study.^30^ In this study, patients received 2 cycles of nivolumab with ipilimumab, following which they underwent resection of an ILN. Patients who experienced a mPR did not undergo TLND or receive further systemic therapy, patients with partial response underwent TLND but no further systemic therapy, and patients with pNR underwent TLND and received adjuvant therapy. The 2-year RFS rate for patients who experienced mPR with neoadjuvant therapy was 93%. The NADINA trial reported estimated RFS at 12 months of 95.1% for patients who had an mPR following neoadjuvant therapy, though follow-up data for this trial is relatively immature. An increasing body of data support the utility of pathological response to neoadjuvant therapy as a prognostic biomarker in melanoma. In a pooled analysis of 6 clinical trials assessing neoadjuvant therapy in melanoma, patients who experienced a pCR with neoadjuvant therapy had a superior RFS than patients without pCR^15^. While a response-adapted treatment strategy is increasingly attractive based on NADINA trial data, coupled with the emerging validity of pCR and mPR as prognostic biomarkers, longer-term follow-up was felt by group members to be important to confirming the safety and efficacy of this approach.

### Response assessment and operative considerations

#### Radiologic response assessment

High levels of consensus were achieved for statements 16 and 17, which address the timing and imaging modalities for response assessment after neoadjuvant therapy. A pooled analysis of neoadjuvant trials of ICI and targeted therapy in melanoma reported an objective response rate (ORR) of 64% across studies by RECIST criteria.^31^ Most patients (83%) with complete radiologic response had a pCR or near-pCR at the time of surgical resection, while 92% of patients with radiologic disease progression were pathologic non-responders, indicating correlation between radiologic and pathologic response.^15^ Timely radiologic assessment of response to neoadjuvant therapy is necessary to identify the small minority of patients experiencing disease progression precluding definitive resection.^17^^,^^18^

#### Pathologic response assessment

High levels of consensus were achieved for statements 22 and 23 which outline the preferred approach to pathologic response assessment in line with INMC guidelines. MDM discussion highlighted workload implications of the technical handling and sectioning requirements of this approach, particularly if multiple lymph nodes are involved. Workforce planning for pathology departments was viewed as integral to the adoption of this approach. The INMC consensus guidelines recommend serial sectioning at 3-4 mm intervals with entire nodal specimen submission for lymph nodes ≤5 cm in greatest dimension.^32^ For lymph nodes >5 cm, representative sections with the same sectioning intervals are sufficient. Quantifying pathologic response in this manner has important prognostic implications for patients following definitive resection of nodal disease, and strong inter-observer reproducibility has been demonstrated.^33^

#### Operative considerations and extent of nodal resection

High levels of consensus were achieved for our consensus statement which proposes that surgical resection take place within 4-6 weeks of completing neoadjuvant therapy. Statement 19, which outlines TLND as the standard surgical approach for clinically detectable stage III melanoma did not meet criteria for consensus, with 71% and 73% of respondents agreeing with this statement for rounds 1 and 2 of the Delphi process, respectively (see Table 5). Consensus was achieved for statements 20 and 21 which allow consideration of more limited, selective dissection of involved nodal disease after neoadjuvant systemic therapy. Statement 21 emphasizes the importance of informed discussion with the patient regarding potential risks and limitations of adopting a modified surgical approach.

The standard approach for stage III melanoma with macroscopic nodal disease prior to the era of adjuvant or perioperative immunotherapy has been TLND of the involved nodal basin.^23^ Given the potential morbidity associated with TLND, omission of this procedure or minimizing the extent of resection for select patients is attractive for oncologic surgeons, particularly in TLND of the groin nodal basin where chronic lymphedema rates of up to 30% have been reported in retrospective studies.^34^ Retrospective analysis from the patients included in the OpACIN and OpACIN-neo trials who underwent ILN marker placement pre-operatively demonstrated 99% concordance in pathological response between ILN sampling and TLND.^35^ However, the only prospective trial data that assessed the omission of TLND on the basis of pathological response is from the PRADO expansion cohort of the OpACIN-neo study which is significantly limited by sample size and lack of randomization.^24^ The lack of consensus on this topic reflects the difficulty in determining the optimal surgical approach to clinical stage III melanoma in the era of neoadjuvant therapy.

## Discussion

Our modified Delphi study was conducted to critically appraise the evidence supporting perioperative immunotherapy for clinically detectable stage III melanoma amenable to definitive surgical resection. While consensus was achieved for most recommendations, a lack of consensus was identified in key areas including the use of neoadjuvant ICI to down-stage unresectable or borderline-resectable disease, the use of a response-adapted approach to adjuvant therapy and the optimal extent of nodal resection for stage III melanoma. It is noteworthy that an initial analysis of the NADINA trial was reported after the conduct of our Delphi process, reflecting the complexity in interpretation of rapidly evolving evidence in this space for care providers. Local practice for perioperative therapy in this patient population remains perioperative pembrolizumab per the SWOG S1801 regimen, in part because the NADINA approach has not been granted approval for public reimbursement within the local healthcare system.

Addressing the knowledge gaps identified in this process poses a significant challenge for care providers. The use of systemic therapy to downstage potentially resectable disease in the first instance requires more universally accepted criteria for defining resectability, which is currently lacking in the field of melanoma surgery.^23^ Developing internationally recognized criteria for resectability and borderline resectability would help to standardize the prospective assessment of systemic therapy to downstage “borderline” disease.^36^ The optimal extent of nodal dissection for stage III melanoma in the era of perioperative therapy also remains unclear. Given the prognostic utility of pathological response to neoadjuvant therapy, utilizing therapeutic response in an ILN to guide the extent of surgical resection for clinically detectable nodal disease, is attractive mechanistically. The ongoing prospective, cohort EXCILYNT and MELCONSURG studies hope to inform the minimum safe nodal resection required for patients with clinical stage III disease.^37^^,^^38^ The phase III NADINA trial offers the highest level of evidence to date in support of a response-adapted approach to adjuvant therapy.^18^

Our study was potentially limited by the single center approach and suboptimal response rates to the circulated survey. Moreover, individual responses from our MDM members remained anonymized throughout—a standard approach in many Delphi processes. Allowing identification of responses from surveyed experts may have provided insights into the viewpoints of different specialities. The consensus statements and evidence appraisal generated as part of this study will be used to develop a clinical pathway for the implementation of neoadjuvant immunotherapy in melanoma and to guide resource allocation and workforce planning to ensure its effective implementation. We plan to expand our Delphi process to include other Irish centers involved in the multidisciplinary management of melanoma, with an emphasis on incorporating up-to-date published data and improving participant response rates to strengthen the consensus recommendations generated.

## Data Availability

The data presented in this study are available on request from the corresponding author.
